# Can Lipoic Acid Attenuate Cardiovascular Disturbances Induced by Ethanol and Disulfiram Administration Separately or Jointly in Rats?

**DOI:** 10.1155/2019/1974982

**Published:** 2019-11-22

**Authors:** Anna Bilska-Wilkosz, Magdalena Kotańska, Magdalena Górny, Barbara Filipek, Małgorzata Iciek

**Affiliations:** ^1^Chair of Medical Biochemistry, Jagiellonian University, Medical College, 7 Kopernika Street, PL 31-034 Kraków, Poland; ^2^Department of Pharmacodynamics, Jagiellonian University, Medical College, 9 Medyczna Street, PL 30-688 Kraków, Poland

## Abstract

The exogenous lipoic acid (LA) is successfully used as a drug in the treatment of many diseases. It is assumed that after administration, LA is transported to the intracellular compartments and reduced to dihydrolipoic acid (DHLA) which is catalyzed by NAD(P)H-dependent enzymes. The purpose of this study was to investigate whether LA can attenuate cardiovascular disturbances induced by ethanol (EtOH) and disulfiram (DSF) administration separately or jointly in rats. For this purpose, we measured systolic and diastolic blood pressure, recorded electrocardiogram (ECG), and estimated mortality of rats. We also studied the activity of aldehyde dehydrogenase (ALDH) in the rat liver. It was shown for the first time that LA partially attenuated the cardiac arrhythmia (extrasystoles and atrioventricular blocks) induced by EtOH and reduced the EtOH-induced mortality of animals, which suggests that LA may have a potential for use in cardiac disturbance in conditions of acute EtOH intoxication. The administration of EtOH, LA, and DSF separately or jointly affected the ALDH activity in the rat liver since a significant decrease in the activity of the enzyme was observed in all treatment groups. The results indicating that LA is an inhibitor of ALDH activity are very surprising.

## 1. Introduction

According to the World Health Organization (WHO) in 2016, the alcohol abuse resulted in 3 million deaths (5.3% of all deaths) worldwide and 132.6 million disability-adjusted life years (DALYs), i.e., 5.1% of all DALYs in that year [1]. DALYs are the sum of years of life lost due to premature mortality as well as years of life lost due to time lived in less than full health.

According to the authors of that report, the cardiovascular diseases (CVDs) are the leading cause of mortality globally, causing 17.9 million deaths (31.6% of all deaths) and 413.2 million DALYs (15.9% of all DALYs). Globally in 2016, alcohol caused an estimated net CVD burden of 593 000 deaths (3.3% of all CVD deaths) and 13 million CVD DALYs (3.2% of all CVD DALYs). CVDs were responsible for 19.8% and 9.8% of all alcohol-attributable deaths and DALYs lost, respectively [1].

Metabolism of ethanol (EtOH) in the human body occurs mainly in the liver. EtOH can be oxidized to acetaldehyde by three routes: (1) in the presence of NAD in the reversible reaction catalyzed by alcohol dehydrogenase (ADH; E.C 1.1.1.1), (2) in the presence of NADPH and molecular oxygen (O_2_) in the reaction catalyzed by microsomal ethanol oxidizing system (MEOS), and (3) in the presence of hydrogen peroxide (H_2_O_2_) in the reaction catalyzed by catalase (EC 1.11.1.6). ADH is the main enzyme in EtOH metabolism. This enzyme oxidizes 92–96% of the ingested alcohol [2].

(1) $$\begin{array}{c} {\text{C}}_{2}H_{5}OH+{NAD}^{+}\overset{ADH}{⟷}{CH}_{3}CHO+NADH+H^{+}\\ {\text{C}}_{2}H_{5}OH+NADPH+H^{+}+O_{2}\overset{MEOS}{\to }{CH}_{3}CHO+{NADP}^{+}+2H_{2}O\\ {\text{C}}_{2}H_{5}OH+H_{2}O_{2}\overset{catalase}{\to }{CH}_{3}CHO+2H_{2}O \end{array}$$

The second step of EtOH metabolism is catalyzed by aldehyde dehydrogenase (ALDH; E.C 1.2.1.3). This enzyme converts acetaldehyde to acetic acid which can be involved in a number of metabolic processes within the organism.

(2) $$\begin{array}{c} {CH}_{3}CHO+{NAD}^{+}\overset{ALDH}{\to }{CH}_{3}COOH+NADH+H^{+} \end{array}$$

Disulfiram (tetraethylthiuram disulfide, antabuse, or DSF) is a well-known inhibitor of ALDH. By inhibiting ALDH activity, DSF causes EtOH intolerance due to poisoning with acetaldehyde, the concentration of which is high after EtOH consumption. It means that EtOH intake during DSF treatment results in the accumulation of acetaldehyde, which is associated with a risk of severe often life-threatening cardiovascular disturbances, including cardiac arrhythmia, drop in blood pressure, circulatory collapse, and death. These unpleasant and potentially life-threatening symptoms are known as DSF-EtOH reaction (DER). Of course, it is known that intoxication by excessive EtOH consumption alone (without DSF) can also cause death due to hypotonia and cardiovascular failure [3].

Therefore, it appears that it is justified to search for drugs capable of attenuating toxicity of EtOH alone and preventing or halting the progression of the DER/DSF-like reactions. We wished to examine whether lipoic acid (LA, 5-[(3R)-dithiolan-3-yl]pentanoic acid) would be able to attenuate cardiovascular disturbances induced by EtOH and/or DSF. Why LA? There are reports that LA is able to reduce myocardial injury and preserve cardiac function during ischemia-reperfusion injury [4, 5]. Many studies on animal models have also confirmed that LA can prevent progressive remodeling and even improve cardiac function [6]. Dudek et al. have indicated that LA protects the heart against myocardial post ischemia-reperfusion arrhythmias via K_ATP_ channel activation in isolated rat hearts [7]. The experimental study of Sokołowska et al. has proven a beneficial effect of LA on cyanate toxicity in the rat heart [8]. Skibska et al. in their excellent review indicated that although LA is used in various diseases, it can be particularly effective in cardiovascular diseases, including ischemic heart disease, hypertension, and heart failure [9].

Thus, in this study, we hypothesized for the first time that LA treatment can attenuate cardiovascular disturbances induced by EtOH and DSF administration separately or jointly in rats. For this purpose, systolic and diastolic blood pressure were measured, electrocardiogram (ECG) was recorded, and mortality of rats was estimated. Moreover, the activity of ALDH in the rat liver was studied.

## 2. Materials and Methods

### 2.1. Reagents

In this study, the formulation Thiogamma was used, which contains LA as the pharmacologically active substance. Thiogamma was obtained from Hexal®AG, (Holzkirchen, Germany). Thiopental sodium was obtained from HEFA-Freon Arzneimittel (Germany). Heparin sodium was obtained from Polfa S.A. (Poland). Disulfiram, EDTA, Folin-Ciocalteau reagent, 4-methylpyrazole, NAD, propionaldehyde, and rotenone were provided by Sigma-Aldrich Chemical Company (Poznań, Poland). All other reagents were of analytical grade and were obtained from Polish Chemical Reagent Company (POCh, Gliwice, Poland).

### 2.2. Animals and In Vivo Treatment

All procedures were performed according to the Animal Use and Care Committee Guidelines and were approved by the Ethics Committee of the Jagiellonian University, Kraków, Poland (registration number 106/2009 and ZI/UJ/403/2007). The experiments were carried out on male Wistar rats (180–200 g). Animals were housed in plastic cages in a room at a constant temperature of 20 ± 2°C with a natural light-dark cycle. They had free access to standard pellet diet and water. The tested compounds were administered at doses calculated per kg body weight (bw). EtOH was given by a gavage, and LA and DSF by intraperitoneal (IP) injection. The control group was injected IP with 0.6 ml of saline (0.9% NaCl).

### 2.3. Blood Pressure

The rats were anesthetized by IP injection of thiopental (70 mg/kg). The left carotid artery was cannulated with polyethylene tubing filled with heparin solution in saline to facilitate pressure measurements using a Datamax apparatus (Columbus Instruments, Ohio). The animals were assigned to eight groups of 6 animals each. Blood pressure was measured as follows:

- Group received 0.9% NaCl: measurement lasted 30 min
- EtOH group: measurement was conducted before EtOH administration (2.5 g/kg) and was continued for 30 min thereafter
- Group I (LA group): measurement was conducted before LA administration (100 mg/kg) and was continued for 60 min thereafter
- Group II (DSF group): measurement was conducted before DSF (100 mg/kg) administration and was continued for 60 min thereafter
- Group III (DSF+LA group): measurement was conducted before LA (100 mg/kg) and DSF (100 mg/kg) administration and was continued for 60 min thereafter
- Group IV (LA+EtOH group): measurement was conducted before LA administration (100 mg/kg) and was continued for 30 min thereafter. In the 30^th^ min, EtOH (2.5 g/kg) was administered and the measurement was continued for the next 30 min
- Group V (DSF+EtOH group): measurement was conducted before DSF (100 mg/kg) administration and was continued for 30 min thereafter. In the 30^th^ min, EtOH (2.5 g/kg) was administered and the measurement was continued for the next 30 min
- Group VI (LA+DSF+EtOH group): measurement was conducted before LA (100 mg/kg) and DSF (100 mg/kg) administration and was continued 30 min thereafter. In the 30^th^ min, EtOH (2.5 g/kg) was administered and the measurement was continued for the next 30 min

Subsequently, animals were sacrificed. The livers were removed, washed in 0.9% NaCl, placed in liquid nitrogen, and stored at -80°C until biochemical tests were performed.

### 2.4. Electrocardiogram Recording (ECG)

The rats were anesthetized by IP injection of thiopental at a dose of 70 mg/kg. The ECG was recorded with three needle electrodes placed on the skin on the right and left chest and the left foot. Electrocardiographic investigations were carried out using Multicard 30 apparatus, standard lead II, and paper speed of 50 mm/s. The ECG was standardized before each tracing to achieve appropriate sensitivity (2 mV pulse produces 20 mm height) and chart speed (50 mm/s). The animals were assigned to seven groups of 6 animals. ECG evaluated such parameters as extrasystole, bradycardia, atrioventricular blocks, and mortality. For each group, the percentage of animals, in which the disturbance occurred, was calculated.

- Group A (EtOH): the ECG recording started in the 1^st^ min and ended in the 30^th^ min after EtOH administration (2.5 g/kg)
- Group B (LA+EtOH): the ECG recording started in the 1^st^ min after LA administration (100 mg/kg) and was conducted for the next 30 min. In the 30^th^ min, EtOH (2.5 g/kg) was administered; the ECG recording ended in the 60^th^ min after LA administration (30 min after EtOH administration)
- Group C (LA): the ECG recording started in the 1^st^ min and ended in the 60^th^ min after LA administration (100 mg/kg)
- Group D (DSF): the ECG recording started in the 1^st^ min and ended in the 60^th^ min after DSF administration (100 mg/kg)
- Group E (LA+DSF group): the ECG recording started in the 1^st^ min and ended in 60^th^ min after LA (100 mg/kg) and DSF (100 mg/kg) administration
- Group F (LA+DSF+EtOH group): the ECG recording started in the 1^st^ min after LA (100 mg/kg) and DSF (100 mg/kg) administration and was conducted for the next 30 min. In the 30^th^ min, EtOH (2.5 g/kg) was administered. The recording of ECG ended in the 60^th^ min after LA and DSF administration (30 min after EtOH administration)
- Group G (DSF+EtOH group): the ECG recording started in the 1^st^ min after DSF (100 mg/kg) administration and was conducted for the next 30 min. In the 30^th^ min, EtOH (2.5 g/kg) was administered. The ECG recording ended in the 60^th^ min after DSF administration (30 min after EtOH administration)

### 2.5. Determination of ALDH Activity in the Rat Liver Homogenate

The assay mixture contained liver homogenate, sodium phosphate buffer (pH 8.2), NAD, EDTA, 4-methylpyrazole, and rotenone. The reaction was initiated by the addition of propionaldehyde as a substrate. 4-Methylpyrazole was added to inhibit alcohol dehydrogenase, and rotenone to inhibit mitochondrial NADH oxidase. The blank sample without the homogenate or the substrate was run simultaneously. The activity of ALDH was calculated by using the molar extinction coefficient of NADH of 6.22 mM^−1^ cm^−1^ at 340 nm with the use of a modified protocol published earlier [10, 11]. Enzyme-specific activity was expressed as nmol NADH min^−1^ mg^−1^ protein. The protein content was determined by the method which is based on the reaction of peptide bonds and aromatic amino acid residues with Folin-Ciocalteau reagent (a mixture of phosphotungstic and phosphomolybdic acids) in alkaline environment in the presence of copper (II) ions. Protein bound with copper ions reduces the above acids to respective oxides. Absorbance was measured at 500 nm [12].

### 2.6. Statistics

All statistical calculations were carried out with the use of GraphPad Prism 6.0 computer program.

The results obtained from measurements of blood pressure are presented as the mean ± SEM of animals in each group. The statistical calculations were performed using a two-way ANOVA Multiple Comparison and post hoc Tukey test.

The results obtained from measurements of ALDH activity in rat liver homogenates are presented as the mean ± SD for each group of animals. The statistical calculations were performed using a one-way ANOVA and post hoc Tukey test.

For all data, the values of *p* < 0.05 were considered to be statistically significant.

## 3. Results

### 3.1. The Effect of Ethanol Administration on Systolic and Diastolic Blood Pressure in Rats

**Figure 1:**
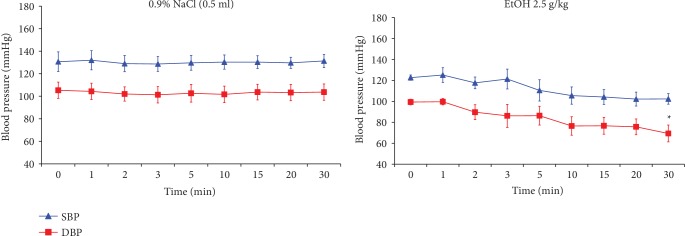
The effect of 0.9% NaCl or EtOH on the systolic and diastolic blood pressure (SBP and DBP, respectively) in rats measured for 30 min. Each group comprised 6 animals (*n* = 6). Data are shown as the mean ± SEM. Significant *vs.* the blood pressure measured before the administration of EtOH, i.e., at *t* = 0; ^∗^*p* < 0.05.

The blood pressure in rats, which received 0.9% NaCl was 130.67/105.33 mmHg at *t* = 0 measurement. The blood pressure was 105.33/103.67 mmHg measured at 30 minutes (*t* = 30) after administration of salt (Figure 1).

EtOH administration caused a statistically significant decrease only in diastolic blood pressure at 30 minutes of measurement compared to the pressure measured before the administration of EtOH, i.e., at *t* = 0 (Figure 1). Blood pressure measured before EtOH administration (*t* = 0) was 122.67/99.33 mmHg, while measured at 30 min (*t* = 30) amounted to 102.33/69.33 mmHg (Figure 1).

### 3.2. The Effect of Lipoic Acid on the Systolic and Diastolic Blood Pressure in Rats

**Figure 2:**
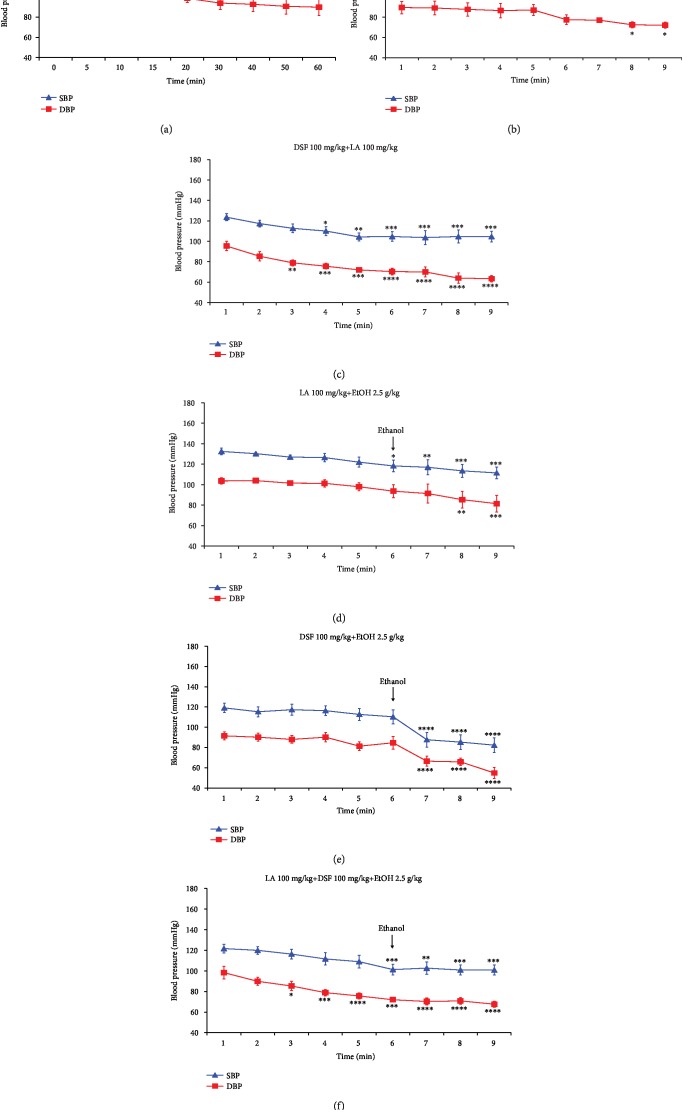
The effect of LA, DSF, and various combinations of these chemical compounds with EtOH on the systolic and diastolic blood pressure (SBP and DBP, respectively) in rats. Each group comprised 6 animals (*n* = 6). Data are shown as the mean ± SEM. Significant *vs.* the blood pressure measured at *t* = 0 minutes, i.e., before the administration of any drugs. ^∗^*p* < 0.05; ^∗∗∗^*p* < 0.001.

Systolic blood pressure measured before administration of LA (*t* = 0) was 132.5 mmHg, while measured 60 minutes after administration of LA was 115.33 mmHg. Statistically significant changes in systolic blood pressure were observed from 30 minutes after administration of LA. This means that LA lowers systolic blood pressure in rats. However, the effect of LA on the diastolic blood pressure was not observed (Figure 2(a)).

### 3.3. The Effect of Disulfiram on the Systolic and Diastolic Blood Pressure in Rats

DSF administration statistically significantly decreased systolic and diastolic blood pressure in rats. Blood pressure measured before DSF treatment (*t* = 0) was 127.67/89.5 mmHg while determined at 60 minutes (*t* = 60) was 105/72 mmHg. Statistically significant changes in systolic and diastolic blood pressure were observed from 40^th^ to 50^th^ minute, respectively, after administration of DSF. This means that DSF lowers blood pressure in rats (Figure 2(b)).

### 3.4. The Effect of Combined Lipoic Acid and Disulfiram Treatment on the Systolic and Diastolic Blood Pressure in Rats

The combined LA and DSF treatment statistically significantly reduced systolic and diastolic blood pressure in rats. Blood pressure before joint administration of LA and DSF (*t* = 0) was 123.6/95.33 mmHg, while recorded at 60 minutes (*t* = 60) was 104.67/63.33 mmHg (Figure 2(c)). Statistically significant changes in diastolic and systolic blood pressure were observed from 10^th^ to 15^th^ minute, respectively, after administration of LA and DSF.

This means that the combined administration of LA and DSF lowers blood pressure in rats (Figure 2(c)).

### 3.5. The Effect of Combined Lipoic Acid and Ethanol Treatment on the Systolic and Diastolic Blood Pressure in Rats

The combined LA and EtOH treatment statistically significantly reduced systolic and diastolic blood pressure in rats. Blood pressure before administration of EtOH was 118.33/93.67 mmHg, while measured 30 minutes after administration of EtOH was 111.5/81.5 mmHg. However, the pressure measured at *t* = 0 minutes, i.e., before any compounds were administered, was 132.5/103.67 mmHg (Figure 2(d)).

Blood pressure values measured in the EtOH group, LA group, and LA+EtOH group at the endpoint of the experiment were 102.33/69.33, 115.33/89.67, and 111.5/81.5, respectively.

This means that in the presence of LA, the decrease in blood pressure is smaller than in the group of rats receiving only EtOH.

### 3.6. The Effect of Combined Disulfiram and Ethanol Treatment on the Systolic and Diastolic Blood Pressure in Rats

The combined DSF and EtOH treatment statistically significantly reduced systolic and diastolic blood pressure in rats. Blood pressure before administration of EtOH was 110.33/84.67 mmHg, while measured 30 minutes after administration of EtOH was 82.33/54.87 mmHg. However, the pressure measured at *t* = 0 minutes, i.e., before any compounds were administered, was 119.17/91.5 mmHg (Figure 2(e)).

Blood pressure values measured in the EtOH group, DSF group, and DSF+EtOH group at the endpoint of the experiment were 102.33/69.33, 105/72, and 82.33/54.87 mmHg, respectively.

This means that in the presence of DSF, the decrease in blood pressure is bigger than in the group of rats receiving only EtOH.

### 3.7. The Effect of Combined Lipoic Acid, Disulfiram, and Ethanol Treatment on the Systolic and Diastolic Blood Pressure in Rats

Blood pressure before administration of EtOH was 101.33/72 mmHg, while measured 30 minutes after administration of EtOH was 100.87/67.67 mmHg. However, the pressure measured at *t* = 0 minutes, i.e., before LA and DSF were administered, was 121.67/98.33 mmHg (Figure 2(f)).

Blood pressure values measured in the EtOH group, LA group, DSF group, LA+DSF group, LA+EtOH group, DSF+EtOH group, and LA+DSF+EtOH at the endpoint of the experiment were 102.33/69.33, 115.33/89.67, 105/72, 104.67/63.33, 111.5/81.5, 82.33/54.87, and 100.87/67.67 mmHg, respectively.

This means that the greatest reduction in blood pressure occurs in the group of rats receiving DSF and EtOH (82.33/54.87 mmHg). However, in the group of animals, which apart from DSF and EtOH also received LA, the decrease in blood pressure was significantly smaller (100.87/67.67 mmHg).

**Figure 3:**
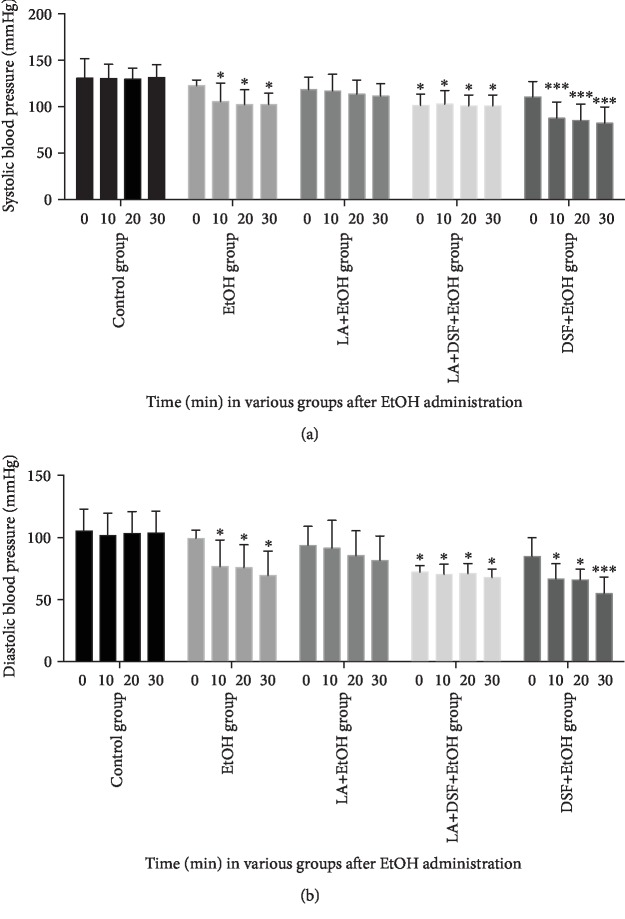
The changes in systolic (a) and diastolic (b) blood pressure in all groups of rats which received EtOH. Each group comprised 6 animals (*n* = 6). Data are shown as the mean ± SEM. Significant *vs.* group receiving 0.9% NaCl (control group); ^∗^*p* < 0.05; ^∗∗∗^*p* < 0.001.

Figure 3 shows changes in systolic (a) and diastolic (b) blood pressure in all groups of rats which received EtOH. Taken together, a statistically significant decrease in systolic and diastolic blood pressure vs. group receiving only 0.9% NaCl (control group) presented in Figure 1 was observed in 3 groups: EtOH group, DSF+EtOH group, and LA+DSF+EtOH group. The greatest drop in blood pressure vs. group receiving 0.9% NaCl (control group) was noted in EtOH-treated rats which were given DSF 30 min before EtOH.

### 3.8. The Effect of Various Combinations of Ethanol, Lipoic Acid, and Disulfiram on the Heart Rhythm Disturbances in Rats and on the Activity of Aldehyde Dehydrogenase in the Rat Liver

The obtained results are shown in Tables 1 and 2.

Bradycardia was seen in all groups of rats; however, in EtOH-treated group (group A) and in the group treated with EtOH 30 min after LA and DSF administration (group F), bradycardia affected 100% of animals. In the remaining groups, 83.5% of rats exhibited bradycardia. It is noteworthy that bradycardia occurred also in 83.5% of animals receiving LA alone (group C). Moreover, in rats treated first with LA and 30 min later with EtOH (group B), bradycardia was seen also in 83.5% of animals, which could suggest a protective action of LA (Table 1).

Extrasystoles were observed in 50% of rats in the EtOH-treated group (group A) and in 33.3% of rats in the group treated with EtOH 30 min after LA administration (group B). It indicates that prior LA administration reduced the number of animals in which extrasystoles were observed (Table 1).

Atrioventricular blocks were observed in 16.7% of rats receiving EtOH (group A) and in 16.7% of rats treated with EtOH 30 min after LA (group B). In this case, LA administration did not decrease the number of rats with this disturbance (Table 1).

Deaths of animals were observed in four groups. In the EtOH-treated group (group A), four animals died (66.7%), one at 5 min and three at 10 min after EtOH administration. In the LA+EtOH group (group B), one animal died (16.7%) 10 min after EtOH administration (at 40 min of the experiment, EtOH was administered 30 min after LA treatment). In the DSF+LA+EtOH group (group F), four animals died (66.7%), two 10 min after EtOH administration (at 40 min of the experiment) and two 20 min after EtOH administration (at 50 min of the experiment, EtOH was administered 30 min after LA and DSF treatment). In the DSF+EtOH group (group G), four animals died (66.7%) 10 min after EtOH administration (at 40 min of the experiment, EtOH was administered 30 min after DSF).

Taken together, the results presented in Table 1 demonstrated bradycardia in all tested animals (groups A-G), while extrasystoles and atrioventricular blocks were observed only in the group treated with EtOH alone (group A) and in EtOH-treated rats which were given LA 30 min before EtOH (group B). Deaths of animals were observed in all EtOH-treated groups, but the fewest animals died (one animal, which corresponds to 16.7%) in the group of animals which was treated with LA 30 min prior to EtOH administration (group B: LA+EtOH group).

The results presented in Table 2 indicated that the administration of EtOH, LA, and DSF separately or jointly affected the ALDH activity in the rat liver. A significant decrease in the activity of the enzyme vs. group receiving 0.9% NaCl was observed in the all treatment groups.

## 4. Discussion

The obtained results indicated that the greatest blood pressure drop was observed in the group administered EtOH after DSF treatment (Figures 2(e) and 3). It is commonly known that DSF has been used for over 70 years as an alcohol-aversive agent in the treatment of alcoholism. By inhibiting ALDH activity, DSF causes EtOH intolerance due to poisoning with acetaldehyde. It causes a spectrum of undesirable symptoms collectively called DSF-EtOH reaction (DER). Prancheva et al. described a case of a 53-year-old man in whom DER manifested as severe hypotension (blood pressure was 35/20 mmHg) accompanied by ischemic stroke [13].

The present data demonstrated also that in this experiment, a number of animals died in all EtOH-treated groups, but the fewest animals died (one animal, which corresponds to 16.7%) in the LA+EtOH group. The question arises why, despite the relatively low dose of EtOH, 66.7% of the animals died in the group that only received EtOH. It is assumed that blood alcohol levels (BACs) exceeding 500 mg% can result in death in humans as a result of paralysis of respiratory centers in the brain stem. On the other hand, there are known cases when alcohol produced death in humans at the values of BAC ranging from under 200 mg% to over 600 mg% [14, 15]. Why some people survive high BAC and others do not is an open question. In our opinion, it is similar in animals. Jones proposed to consider that when examining deaths due to acute ethanol poisoning, among all other factors, lady luck should not be forgotten [15].

The present data demonstrated also that mortality in the group treated with DSF and then with EtOH (DSF+EtOH) was 66.7% (and was the same as in the group administered LA and DSF before EtOH (LA+DSF+EtOH)). Development of cardiovascular collapse in patients with DER has been described by many authors. Ho et al. reported a patient with DER in whom initial volume resuscitation and dopamine infusion failed to restore the BP and blood pressure returned to normal range only after noradrenaline administration [16]. A similar case was described by Milne and Parke [17]. The present results indicate that LA can partially attenuate arrhythmia (extrasystoles and atrioventricular blocks) induced by EtOH. Many literature data confirm that LA exerts powerful protective effects in numerous cardiovascular diseases [7, 18, 19].

The obtained results demonstrated also that all DSF-treated groups of animals showed the reduced ALDH activity in the liver which is obvious (Table 2). In addition, we observed that EtOH given alone also inhibited ALDH activity (Table 2). Despite the counter-intuitive nature of this discovery, the result is consistent with some results obtained by other authors [20–22]. On the other hand, the results indicating that LA is an inhibitor of ALDH activity turned out to be very surprising (Table 2), the more so that our previous observations in vitro indicated that the presence of LA or DHLA had no effect on the activity of yeast ALDH. Those observations demonstrated also that DHLA but not LA could protect ALDH against DSF-induced blockade, while neither LA nor DHLA were able to restore the ALDH activity when it already had been inhibited by DSF [23]. However, it should be noted that it is increasingly more frequently underlined that biological effects of LA obtained in vitro often differ from those observed in vivo which was largely unnoticed until recently [24, 25]. Some authors highlighted that LA could participate, like glutathione (GSH), in S-thiolation of proteins and this process is perceived to be a molecular “switch” decisive for intracellular redox status of thiols able to control cellular metabolic pathways [25]. In the case of GSH, the formation of a mixed disulfide (G-S-S-Protein) usually leads to inhibition of the biological activity of the protein [26].

The mammalian ALDH is a redox-sensitive protein, and the cysteine sulfhydryl group in its active site plays an essential role in its activity. Therefore, it is possible that ALDH forms a mixed disulfide with LA (LA-S-S-ALDH) which leads to a decrease in its activity. However, the problem of S-thiolation involving LA is still not well understood and there is virtually no literature data while those that can be found are based more on suppositions and deductions, and not on hard scientific evidence. Therefore, it seems that this direction of research is worthy of attention.

Taken together, the present results indicated that LA in rats can attenuate cardiovascular disorders induced by the administration of EtOH. In our opinion, it was caused by the ability of LA to ameliorate the reductive stress which occurs as a result of metabolic processes related to EtOH. The reductive stress is a counterpart of oxidative stress and is defined as an abnormal increase in reducing equivalents. EtOH produces reductive stress since its metabolism results in overproduction of NADH. A decrease in the NAD^+^/NADH ratio causes a decline in the activity of oxidoreductases (reductive stress), resulting in an altered metabolic situation that might be the first insult leading towards several pathologies [27]. NADH excess has many metabolic consequences, of which suppression of the Krebs cycle (tricarboxylic acid cycle (TCA cycle)) and the mitochondrial respiratory chain seem the most important. It is known that the TCA cycle together with the associated electron transport systems plays a key role in energy production. Inhibition of both TCA cycle and respiratory chain hinders energy generation from any compound, including EtOH. Thus, EtOH insidiously “deceives” the body because NADH accumulation and TCA cycle inhibition are signals of intracellular energy abundance, whereas actually the body faces a significant energy deficit. Furthermore, the elevated NADH levels suppress synthesis of UDP-glucuronic acid (UDPGA) compounds that must be attached to various xenobiotics (drugs, poisons, dietary supplements, etc.) before they can be excreted from the body.

Some researchers have observed that many degenerative diseases are associated with a hypoxic state that results in an increased NADH/NAD^+^ ratio [28, 29]. Many authors also showed that reductive stress played an important role in the development of cardiomyopathy in mice [30, 31]. In addition, overproduction of reducing equivalents imparted pleiotropic effects on gene expression, mitochondrial dysfunction [32], and protein quality control [33] in cardiomyopathy in mice.

It should be remembered that exogenous LA after administration is reduced to DHLA by NAD(P)H-dependent enzymes which in a simplified form can be expressed as

(3) $$\begin{array}{c} LA+NADH+H^{+}⟶DHLA+{NAD}^{+} \end{array}$$

As a result of this reaction, NADH is oxidized to NAD, and thus the NAD^+^/NADH ratio increases. Hence, it is a plausible proposal that LA partially attenuated the toxicity of EtOH by decreasing the reductive stress level. It is worth noting that this is the first time when the novel hypothesis was presented that LA, which is normally considered to be an antioxidant, is also required for protection against reductive stress.

In conclusion, this study for the first time demonstrated that LA could partially attenuate the cardiac arrhythmia (extrasystoles and atrioventricular blocks) induced by EtOH and reduced the EtOH-induced mortality of animals, which supports a potential of LA for use in acute EtOH-intoxication and suggests that further experiments are necessary to elucidate the mechanism of action of LA as an antidote to EtOH poisoning.

## Figures and Tables

**Table 1:** The effect of various combinations of ethanol (EtOH), lipoic acid (LA), and disulfiram (DSF) on the heart rhythm disturbances and mortality in rats.

Disorders	EtOH	LA+EtOH	LA	DSF	DSF+LA	DSF+LA+EtOH	DSF+EtOH
	Group A	Group B	Group C	Group D	Group E	Group F	Group G
Extrasystoles	3 min (50%)	30 min (33.3%)	None	None	None	None	None
Bradycardia	5 min (100%)	40 min (83.5%)	60 min (83.5%)	20 min (83.5%)	25 min (83.5%)	30 min (100%)	40 min (83.5%)
Atrioventricular blocks (min/%)	3/16.7 5/16.7 30/16.7	30/16.7	None	None	None	None	None
Death (min/%)	5/16.7 10/50	40/16.7	None	None	None	40/33.3 50/33.3	40/66.7

Each group comprised 6 animals (*n* = 6). Data are presented as a percentage of animals with the heart rhythm disturbances and mortality in rats.

**Table 2:** The effect of various combinations of ethanol (EtOH), lipoic acid (LA), and disulfiram (DSF) on the aldehyde dehydrogenase activity in the rat liver.

Treatment	Activity of ALDH in the rat liver (IU/min/mg protein)
Group receiving NaCl	38.41 ± 5.00
EtOH group	^aaa,bbb^18.075 ± 4.05^c^
LA (group I)	^aaa,b^11.3 ± 4.44^d^
DSF (group II)	^aaa^6.43 ± 1.37^ddd^
LA+DSF (group III)	^aaa,ccc^6.75 ± 1.42^ddd^
LA+EtOH (group IV)	^aaa^8.10 ± 2.66^ddd^
DSF+EtOH (group V)	^aaa^6.56 ± 1.04^ddd^
LA+DSF+EtOH (group VI)	^aaa,ccc^4.09 ± 2.00^ddd^

Data are shown as the mean ± standard deviation (SD) of 6 animals per group (*n* = 6); ^aaa^*p* < 0.001 vs. group receiving 0.9% NaCl; ^b^*p* < 0.05 and ^bbb^*p* < 0.001 vs. DSF group; ^c^*p* < 0.05 and ^ccc^*p* < 0.001 vs. LA group, ^d^*p* < 0.05 and ^ddd^*p* < 0.001 vs. EtOH group.

## Data Availability

No data were used to support this study.
